# Bacterial Diversity Profiling of Desert Sand from Tierra Caliente, Mexico, Based on 16S rRNA Gene Amplicon Sequencing

**DOI:** 10.1128/mra.00782-22

**Published:** 2022-08-24

**Authors:** Cristal Ramos-Madrigal, Esperanza Martínez-Romero, Yunuen Tapia-Torres, Luis E. Servín-Garcidueñas

**Affiliations:** a Laboratorio de Microbiómica, Escuela Nacional de Estudios Superiores Unidad Morelia, UNAM, Morelia, Michoacán, México; b Programa de Ecología Genómica, Centro de Ciencias Genómicas, UNAM, Cuernavaca, Morelos, México; University of Delaware

## Abstract

Tierra Caliente is an arid region in Mexico, representing a reservoir of understudied xerophilic bacteria. Here, we report the bacterial amplicon sequencing characterization of desert sand collected from the Zicuirán-Infiernillo Biosphere Reserve within Tierra Caliente. Bacteria known to be resistant to desiccation and high radiation were detected.

## ANNOUNCEMENT

Deserts are one of the most common biomes in the world, covering about a fifth of the surface of the planet (1). Xerophilic microorganisms are extremophiles that can survive and grow with low water availability, conditions present in arid environments, and their study is relevant for understanding resistance mechanisms (2). In Mexico, the Tierra Caliente region contains low-elevation areas characterized by high temperatures and low precipitation. The bacterial diversity of this region has remained unexplored until recently.

A 30-g sample of surface sand was collected from the Zicuirán-Infiernillo Biosphere Reserve (18°46′30″N, 102°0′52″W; 170 m above sea level [masl], 35°C, and 50% humidity), in the region of Tierra Caliente, Michoacán, in October 2021. Sand was collected at a maximum depth of 1 cm using a disinfected and flame-sterilized shovel, sterile gloves, and face masks and stored in sterile 50-mL Falcon tubes. DNA extraction was performed using the DNeasy PowerSoil Pro kit (Qiagen) following the manufacturer’s instructions. The V3 to V4 region of bacterial 16S rRNA genes was amplified using the primers 341F (5′-CCTACGGGNGGCWGCAG-3′) and 805R (5′-GACTACHVGGGTATCTAATCC-3′) (3). The PCR protocol comprised an initial denaturation at 95°C for 3 min, 25 cycles at 95°C for 30 s, annealing at 55°C for 30 s, and extension at 72°C for 30 s, followed by a final extension at 72°C for 5 min. The PCR products were purified using AMPure XP beads (Beckman Coulter, Inc., Fullerton, CA, USA); the Nextera XT index kit (Illumina) was used for library preparation, and the resulting sample was quantified using the Qubit double-stranded DNA (dsDNA) assay kit (Life Technologies, USA). Sequencing was performed using a MiSeq platform with 300-bp paired-end format (Macrogen Co., Seoul, South Korea). The reads were assessed using FastQC v.0.11.8 (with default settings) (4) and were filtered for quality (scores of ≥Q20) and adaptor sequences using Trimmomatic v.0.39 (5). The reads were further processed using the QIIME2 v.2020.8.0 pipeline (6, 7). The q2-dada2 plugin and denoise-single method (8) were used to eliminate noise and chimeras. Amplicon sequence variants (ASVs) were taxonomically assigned using the q2-feature-classifier plugin and the classify-consensus-vsearch method (9) with the SILVA database v.138 SSURef Nr99 (10) as the reference.

The raw sequencing output was 198,556 paired-end reads. After filtering, 98,885 high-quality reads clustered in 1,500 valid ASVs. Taxonomic assignment showed the predominance of the phylum *Actinobacteriota* (35.60%), followed by the phyla *Proteobacteria* (13.77%), *Chloroflexi* (12.95%), *Acidobacteriota* (9.30%), *Firmicutes* (7.07%), *Planctomycetota* (6.22%), *Bacteroidota* (3.54%), and *Gemmatimonadota* (3.13%). This bacterial profile is consistent with those of other arid regions (11–18). The most abundant genera were *Rubrobacter* (7.7%), *Microvirga* (2.26%), *Bacillus* (2.15%), *Solirubrobacter* (1.48%), and *Geodermatophilus* (1.26%), some of which contain bacteria that have been reported to be highly resistant to radiation (19, 20). The data also showed archaeal sequences (fewer than 0.1%) corresponding to the phyla *Thermoplasmatota* and *Crenarchaeota*.

This 16S rRNA gene amplicon sequencing profile is the first reported within this extreme arid region and a valuable resource for future microbial diversity research on Mexican deserts.

### Data availability.

The sequencing data have been deposited in the Sequence Read Archive (SRA) under the accession number SRR19787797 (BioProject accession number PRJNA851896).
